# Experimental Dental Composites Containing a Novel Methacrylate-Functionalized Calcium Phosphate Component: Evaluation of Bioactivity and Physical Properties

**DOI:** 10.3390/polym13132095

**Published:** 2021-06-25

**Authors:** Sunny Skaria, Kenneth J. Berk

**Affiliations:** Pulpdent Corporation, Watertown, MA 02472, USA; ken@pulpdent.com

**Keywords:** dental composite, methacrylated calcium phosphate, esthetic, biomineralization

## Abstract

The aim of this study was to synthesize and characterize a novel methacrylate-functionalized calcium phosphate (MCP) to be used as a bioactive compound for innovative dental composites. The characterization was accomplished by attenuated total reflectance Fourier-transform infrared spectroscopy (ATR-FTIR), scanning electron microscopy (SEM), and energy dispersive spectroscopy (EDS). The incorporation of MCP as a bioactive filler in esthetic dental composite formulations and the ability of MCP containing dental composites to promote the precipitation of hydroxyapatite (HAp) on the surfaces of those dental composites was explored. The translucency parameter, depth of cure, degree of conversion, ion release profile, and other physical properties of the composites were studied with respect to the amount of MCP added to the composites. Composite with 3 wt.% MCP showed the highest flexural strength and translucency compared to the control composite and composites with 6 wt.% and 20 wt.% MCP. The progress of the surface precipitation of hydroxyapatite on the MCP containing dental composites was studied by systematically increasing the MCP content in the composite and the time of specimen storage in Dulbecco’s phosphate-buffered solution with calcium and magnesium. The results suggested that good bioactivity properties are exhibited by MCP containing composites. A direct correlation between the percentage of MCP in a composite formulation, the amount of time the specimen was stored in PBS, and the deposition of hydroxyapatite on the composite’s surface was observed.

## 1. Introduction

Natural bone and teeth are unique composites of biological origin. They consist of a mineral inorganic phase of calcium-phosphates and an organic phase of collagen. Dentin is a mineralized structure that forms the major component of dental hard tissue. It is covered by a highly mineralized and protective layer of enamel in the crown and cementum in the root [1,2].

Demineralization and remineralization processes co-exist in teeth during the entire life of an individual [3]. Dental caries is a dynamic disease process caused by the imbalance of demineralization and remineralization. It occurs in pathological conditions when demineralization outweighs remineralization. The dental caries process begins with the demineralization of apatite by acids produced by oral biofilm bacteria (plaque), followed by the degradation of the extracellular organic matrix of dentin. As caries progresses, degradation of collagen fibrils occurs and leads to a decrease in the mechanical properties of the dentin [4,5]. In general, the caries process is initially a reversible, chronic condition that progresses slowly; it can be arrested by disturbing the plaque biofilm [6].

Remineralization of carious dentin can occur either by a spontaneous incorporation of beneficial ions, such as calcium, phosphate, and fluoride, from the oral fluid into remnant crystallites in the demineralized tissue, or by treatments that incorporate the same beneficial ions from external sources. The repair of dental caries with dental restoratives (fillings) that release calcium, phosphates, and fluoride ions is gaining interest. These bioactive restoratives can help to prevent caries by inducing the remineralization of hypo-mineralized caries lesions and by interfering with the metabolic activity of the biofilm [7,8].

Dental resins containing calcium phosphate fillers such as hydroxyapatite (HAp), amorphous calcium phosphates, nano-calcium phosphates, and mono-, di- and tetra-calcium phosphates have been investigated in recent years as calcium and phosphate releasing composites with remineralizing capabilities [9,10,11,12]. Dental composites containing amorphous calcium phosphate release super-saturated levels of calcium and phosphate ions and have been shown to remineralize tooth lesions in vitro [13,14].

Recently, a combination of reactive calcium dihydrogen phosphate and tri-calcium phosphate has been used. The less soluble tri-calcium phosphate enhances control over the composite’s water absorption and the dissolution of the more soluble calcium dihydrogen phosphate [15]. A light-curable composite containing systematically varying amounts of calcium dihydrogen phosphate, tri-calcium phosphate, and chlorhexidine was investigated for its bioactivity and antibacterial properties [16]. However, the high amounts of calcium phosphate in these formulations reduces the tooth-mimicking esthetics required for successful dental composites, and the low compatibility of the resin phase with the CaP fillers reduces the mechanical properties [17].

Several strategies to increase the interaction of these bioactive fillers with resin phases have been tested [9]. These include (i) silane coupled HAp [18]; (ii) nano-sized amorphous calcium phosphate (NACP) [19,20]; and/or (iii) the functionalization of calcium phosphate particles with dimethacrylate monomers [21,22]. A modest improvement in fracture strength was observed in resin composites containing functionalized fillers in comparison to those containing non-functionalized brushite [23].

A great surge of interest in bioactive dental materials was observed in 2013 after the introduction of the first esthetic, bioactive, dental restorative material, Activa Bioactive-Restorative (AB) (Pulpdent Corporation, Watertown, MA, USA). AB is composed of a reactive, ionic resin (bis-2-(methacryloyloxy)ethyl phosphate) (Bis-2), polycarboxylic acid, and reactive glass. It releases and recharges beneficial calcium, phosphate, and fluoride ions [24], which facilitate remineralization and reduce demineralization of dentinal tissues [25]. In addition to the incorporation of elements for bioactivity, AB materials also contain a rubberized polyurethane-methacrylate-resin that increases fracture toughness and mimics the toughness of dentin [26]. AB facilitates surface precipitation of calcium phosphate, has high tissue compatibility, and induces new osteogenic bone growth [27,28,29]. In these materials, the calcium and phosphate ions are released from the ionomer glass by the reaction of the ionic resin with the ionomer glass.

The original AB materials require a two-part system to derive the benefit of the glass ionomer reaction. To achieve bioactive properties in a one-part material, the authors synthesized methacrylate-functionalized calcium phosphate (MCP), a calcium phosphate with pendent methacrylate groups. Composites incorporating MCP can be formulated as one-part, light-cure only materials. Although dental composites containing varying amounts of bioactive fillers, such as bioglass or calcium phosphate, have been studied, the application of a methacrylate-functionalized calcium phosphate and its commercial development as an esthetic bioactive composite is hitherto unknown. This study investigates the bioactivity of methacrylate-functionalized calcium phosphate containing composites in a concentration dependent manner and the effect of MCP on the optical and physical properties of the composites. The null hypothesis assumed that incorporation of the highly functional methacrylate calcium phosphate (MCP) would not affect the esthetics, translucency, depth of cure, degree of polymerization, and mechanical properties of dental composites.

## 2. Materials and Methods

### 2.1. Materials

#### 2.1.1. Methacrylate-Functionalized Calcium Phosphate (MCP)

Methacrylate-functionalized calcium phosphate (MCP) was synthesized by the conventional solution-precipitation chemistry method; reacting calcium salts with a mixture of phosphoric acid and the phosphate-functional monomer, Bis-2. The reagents calcium chloride dihydrate (Aldrich, Milwaukee, WI, USA); calcium hydroxide (Avantor Performance Materials, Radnor, PA, USA); phosphoric acid 80% (Innophos, Cranbury, NJ, USA); and Bis-2 (Nippon Kayaku, Tokyo, Japan) were used as obtained. The molar ratio of calcium and phosphorous reagents was kept at 1.6. The detailed synthesis of MCP was reported in US Patent [30]. MCP was precipitated from the solution phase by bringing the pH to 10 with the addition of an ammonia solution. The resultant MCP was filtered, washed with distilled water, and dried at room temperature for four days and at 45 °C for two days. The powder was then ball milled for 1 h and sifted. The powder was analyzed by FTIR-ATR, X-ray diffraction spectroscopy (XRD), SEM, and EDS [30]. The Ca/P elemental ratio of the dried MCP powder was determined from colorimetric analysis and was confirmed by the EDS data. The structure of the functional phosphate monomer Bis-2 is presented below.

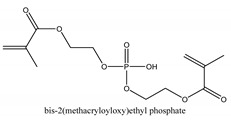



Annealing of the MCP powder was performed in a Paragon E series Kiln equipped with a Sentry 2 Digital Temperature controller (Paragon Industries, Mesquite, TX, USA) by heating 100 mg of MCP powder in a silica crucible to a temperature of 600 °C for 4 h. The weight of the annealed sample was recorded and the amount of the volatile component was determined by the weight difference. The powder turned brown upon annealing, indicating the thermal degradation of the carbonaceus group. This heat treated MCP powder was not used in any of the experimental polymeric formulations, it was made only to calculate the organic content and Ca/P ratio of the hybrid MCP powder.

#### 2.1.2. Synthesis of Polymer Composites

The bioactive ionic resin formulation used in the tested composites contains aliphatic urethane dimethacrylate (UDMA, Evonik Corp., Marl, Germany) and other multi-methacrylate monomers. The composition is proprietary to Pulpdent Corporation, Watertown, MA, USA. The resin mixture also contains a patented rubberized urethane methacrylate resin [31] to provide additional toughness and durability to the composite, and 3 wt.% acidic phosphate monomer (Bis 2), which imparts ionic properties to the resin matrix. The experimental composites were filled with a mixture of radiopaque, silanated barium, and strontium alumino-silicate glasses with an average particle size of 0.7 µm to 4 µm and submicron silica fillers. They contained varying amounts of methacrylate-functionalized calcium phosphate (MCP) as the bioactive filler and 0.4 wt.% other additives (CQ-amine initiator system and colorants). The experimental composite formulations are presented in Table 1. The mixing of the resins and the powders in a batch size of 500 g was carried out in a Ross mixer (Charles Ross & Sons, Hauppauge, NY, USA) at a speed of 80 RPM for 3 h. The formulations were finally shaded to Vita A2 shade to enable an unvarying comparison of the optical and physical properties. The formulations were finally vacuumed and stored in black containers.

To determine the optimum levels of MCP required for bioactivity and esthetics, and to understand the relationship between MCP concentrations in the composite and its bioactivity and mineralization properties, formulations with 3 wt.%, 6 wt.%, and 20 wt.% MCP and a control composite with no MCP were investigated for their ion release and physico-chemical properties. All of the specimens were polymerized by light curing with a light source having an output of 600 mW/cm^2^ (LE Demetron, Kerr, Orange, CA, USA), and the intensity was frequently measured with a hand-held radiometer (Demetron, Kerr, Orange, CA, USA).

### 2.2. Physico-Chemical Characterization

#### 2.2.1. FTIR Characterization

Attenuated total reflection Fourier transform spectroscopy (ATR-FTIR) was employed to characterize the methacrylate-functionalized calcium phosphate (MCP) and the precipitated layer formed on the composite after immersion in PBS. The spectra of the specimens were obtained using a Nicolet IR200 spectrophotometer (Thermo-Fisher, Waltham, MA, USA) with an ATR accessory and were recorded in absorption mode using 4 cm^−1^ resolution with 32 scans and in the range of 500–4000 cm^−1^. The FTIR of the surface precipitated layer was measured by scraping off that layer from the specimens after being stored in phosphate buffered saline (PBS) for 21 days at 37 °C.

#### 2.2.2. Translucency Parameter

The translucency parameter (TP) was calculated from the color coordinate values of the composite material [32]. The color of the composite materials was measured using a reflection spectrophotometer. Specimens 9 mm in diameter and 2 mm in thickness (N = 3) were fabricated in a silicon mold. Each specimen was light cured for 20 s on each side and polished to a thickness of 2 mm on a 600 grit SiC paper measured by a digital micrometer (Mitutoyo, Japan). The color of each specimen was measured according to the CIELAB color scale (color notation system developed by Commission Internationale de l’Eclairage), and related to the standard illuminant D65 against a white background and a black background using a reflection spectrophotometer (Color i7 spectrophotometer, XRite Corp, Grand Rapids, MI, USA). After determining the color of the specimen, the translucency parameter (TP) was calculated by the difference between the color of the specimen over the white background and the color of the specimen over the black background using the following equation:

TP = [(L_w_ − L_b_)^2^ + (a_w_ − a_b_)^2^ + (b_w_ − b_b_)^2^]^1/2^

where L is the coordinate for the lightness; a (green/red) and b (blue/yellow) are chromatic coordinates; the subscript *b* refers to color parameters over the black background; and subscript w refers to color parameters over the white background.

#### 2.2.3. Degree of Conversion (DC)

The degree of conversion of the composites with respect to the amount of the MCP filler in the formulation was evaluated.

Prior to curing, the specimens were fabricated in black silicon molds 1 mm in height and 4 mm in internal diameter placed directly on the ATR cell of the ATR-FTIR spectrophotometer (Nicolet IR 200, Thermo-Fisher, Waltham, MA, USA). A transparent Mylar film was placed over the material, and the spectrum of the specimens was recorded before light activation.

After light curing the specimen from the top surface for 20 s, the spectrum of the bottom layer of each polymerized specimen was scanned. More scans of the bottom layer were recorded after irradiating the specimen at the top surface for 40, 60, and 120 s.

The degree of conversion of the specimens was determined by the ratio of the area of the methacrylate group (C=C) at 1638 cm^−1^ of the polymerized specimen to that of non-polymerized specimen. The DC was calculated according to the following equation [33].

DC (%) = [(A_1_/A_0_ − A_1_′/A_0_′)/A_1_/A_0_)] × 100

where A_1_/A_0_ and A_1_′/A_0_′ are the peak area ratio of methacrylate and amide, before and after polymerization. The amide group of the UDMA at 1537 cm^−1^ was used as the internal standard [33].

#### 2.2.4. Depth of Cure

The depth of cure of each test material was measured by ISO 4049 [34]. Specimens (N = 3) of each material were made in a steel mold 4 mm in internal diameter and 6 mm in height. A transparent Mylar film was placed over it. The specimens were light cured from the top surface for 20 s. After curing, the specimens were immediately removed from the mold and the uncured material at the bottom surface of each specimen was scraped away using a plastic spatula. The height of the remaining specimen was measured using a digital micrometer (Mitutoyo, Kawasaki, Japan). The ISO 4049 mean depth of cure of each material was calculated by averaging the height of the polymerized specimen of each material and dividing the average height of the specimen by two.

#### 2.2.5. Flexural Strength (FS) and Deflection at Break

Six specimen bars with dimensions of 2 × 2 × 25 mm were fabricated for each material by injecting the material into a polypropylene mold. The mold was placed between two glass sides covered with Mylar film. The specimen bars were cured for 1 min on each side using a light source of 600 mW/cm^2^. After curing, the specimen bars were taken out of the mold, excess flash was removed, and the specimens were wet polished with 600 grit paper. The specimens were then stored in PBS for one day at 37 °C. The specimen dimensions were measured by a digital micrometer of 0.01 mm sensitivity (Mitutoyo, Japan). Immediately after removing the specimens from the PBS, the three-point flexural strengths of the specimen bars were tested on a universal testing machine (Instron T1140, Norwood, MA, USA) with a cross head speed of 1 mm/min. The distance between the support beams of the three point jig was 20 mm. Flexural strength (FS) in MPa was calculated using the following equation:

FS = P_Max_ 3L/2bh^2^

where P_Max_ is the load at break, b is the specimen width, h is the specimen thickness, and L = 20.

The deflection at break data for each specimen was generated from the three-point flexural strength measurements and was measured by correlating the crosshead motion to beam deflection.

#### 2.2.6. Ion Release studies

Disk shaped specimens (N = 5) approximately 9 mm in diameter and 2.5 mm in thickness were fabricated by injecting the composite material into a silicon mold and light curing for 20 s on each side. The dimensions of each specimen were then accurately measured using the digital micrometer, and the weight of each specimen was recorded. Each specimen was stored in 10 mL of distilled water (pH 7) in an incubator (Boekel Industries, Feasterville, PA, USA) at 37 °C. After 24 h, each specimen was transferred to a new bottle with 10 mL of fresh distilled water.

The calcium ions present in the solution of the original bottle were measured by UV-Vis colorimetric analysis (Genesis 10S UV-Vis, Thermo Scientific, Waltham, MA, USA), after an aliquot of the solution was treated with cresolphthalein-complexone reagent (Aldrich, Milwaukee, WI, USA) [35]. To calibrate, UV-Vis spectrophotometer standard calcium ion solutions in the concentration ranges of 0.2, 0.4, 1.0, 2.0, 5.0, and 10 ppm were used. The procedure was repeated, and the calcium ion release from the specimens was calculated on days 2, 5, 7, 14, 21, and 28.

The release of phosphate ions was measured by colorimetric analysis using Smart 2 Colorimeter (LaMotte, MD, USA) after complexing with Vanadate Molybdate reagent (Aldrich, Milwaukee, WI, USA). The specimens (N = 5) were stored in 10 mL of distilled water, and the soluble phosphate ions in the solution on day one were determined. The procedure was repeated, and the ion release from the specimens was calculated on days 2, 5, 7, 14, 21, and 28. Calibration curves were generated by 1, 2, 5, 10, 20, and 40 ppm standard solutions of sodium phosphate in distilled water.

#### 2.2.7. Composite Disc Preparation and Bioactivity Studies

The in vitro bioactivity and the bio-mineralization potentials of MCP containing composites were tested in accordance with ISO method 23317:2014 [36] by immersing the specimens in commercial Dulbecco’s phosphate-buffered solution with calcium and magnesium (PBS) (D 8662, Sigma-Aldrich, St. Louis, MO, USA). Thirty cylindrical specimens 9 mm in diameter and approximately 3.6 mm in thickness were prepared in a two-step process: (1) A silicone mold 9 mm in diameter and approximately 1.8 mm thickness was placed on a Mylar sheet on a flat glass plate. The composites were injected into the mold, a nylon thread 6 cm long was placed across the composite, and a Mylar film and glass plate were placed on top. The specimens were light-cured for 20 s (LE Demetron II, Kerr, Orange, CA, USA). (2) The top glass plate and the mylar film were removed, and a second silicon mold of similar dimensions to the first was placed over the cured resin and the nylon thread. The composite material was injected into the second mold. A Mylar film was placed over the material followed by a glass plate. The material was light-cured for another 20 s on each side of the disc (total thickness approximately 3.6 mm). The specimen disc was removed from the mold, its surfaces were polished using a 220-grit SiC paper, and it was washed with distilled water and dried. Using the nylon thread, the specimen discs were suspended vertically in plastic bottles containing 25 mL of PBS and stored at 37 °C. On the 7th day, specimens were removed from the plastic bottles, washed thoroughly with distilled water, dried, and stored in a desiccator for SEM evaluation. The remaining specimens remained in their plastic bottles, and the PBS was changed. This process was repeated for all specimens at 14 days and 21 days.

The specimens were analyzed using SEM (Amray 3300 FESEM, field emission scanning electron microscope) and energy dispersive X-ray spectroscopy (EDS) to assess the morphology and elemental composition of the surface precipitated layer formed on the specimens. Control specimens (0 days) and the specimens stored in PBS for 7, 14, and 21 days were mounted on aluminum SEM stubs using double-sided, carbon adhesive tape. The SEM stubs were sputter-coated with Au-Pd for 90–95 s in a Denton Vacuum Desk II (Moorestown, NJ, USA) to eliminate charging in non-conductive specimens. The specimens were analyzed at 10 KV, condenser spot size −25, objective aperture 200, working distance of 25–28 mm, using a RaySpec light element detector (High Wycombe, UK) for 120–130 s to determine the elemental composition of the material. Images were captured at magnifications of 40×, 100×, 1000×, 2000×, 3000×, and 6000×.

## 3. Results

### 3.1. Synthesis and Characterization of MCP

**Figure 1:**
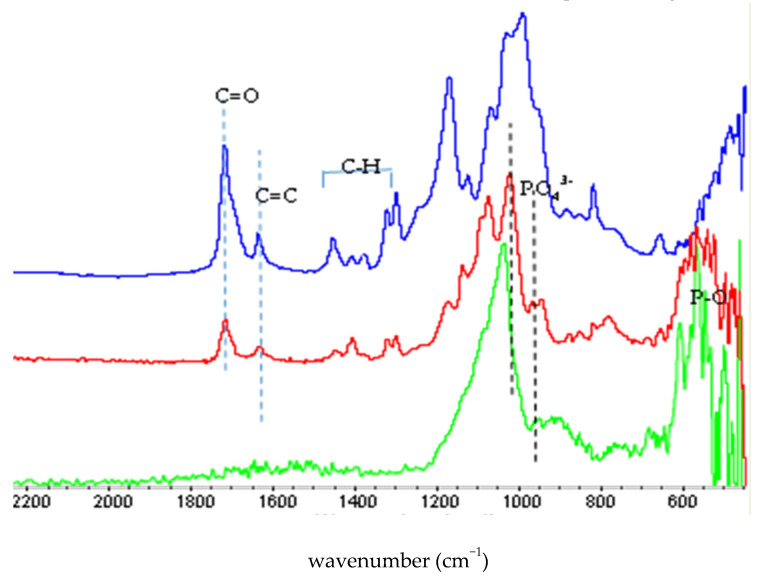
FTIR spectra of Bis 2-(methacryloyloxy) ethyl phosphate (Bis 2) monomer (blue line], methacrylate functionalized calcium phosphate (MCP) (red line), and MCP annealed at 600 °C for 4 h (green line). Both Bis-2 and MCP show the characteristic absorption peak of the methacrylate group (1720 cm^−1^ and 1638 cm^−1^) and other absorption peaks originated from C-O and P-O groups (see text). The annealed MCP showed only absorption peaks characteristic of P-O groups, and no peaks related to the organic functional C-O groups.

The synthesis of methacrylate functionalized calcium phosphate was done according to the patented process [30], and a yield of 90% was obtained. The XRD (presented as a Supplementary file, Figure S1) exhibited broad peaks, characteristic of amorphous or nanocrystalline calcium phosphate. The elemental compositional analysis of the MCP powder showed a Ca/P ratio of 1.24, suggesting that the resultant MCP powders possibly had a compositional ratio of amorphous calcium phosphate. FTIR spectra of the organic functional monomer (Bis-2), the methacrylate-functionalized calcium phosphate powder (MCP), and the annealed MCP (annealed at 600 °C for 4 h) are depicted in Figure 1.

The FTIR spectrum of the MCP powder was very complex. The absorption peaks of MCP powder were close to, or merged with, the absorption peaks of the phosphate monomer (Bis-2), especially in the 950–1300 cm^−1^ region. The absorption peaks of MCP powder at 1720 cm^−1^(C=O) and 1638 cm^−1^ (C=C) indicated that the methacryloyl group was already in the powder. The MCP powder and heat treated MCP powder showed multiple peaks in the 550–605 cm^−1^ region, which were attributed to the bending vibrations of phosphate and hydroxyl groups. The absorption peaks of MCP powder at 1074 cm^−1^ and 1021 cm^−1^ were attributed to the P-O vibration modes of regular tetrahedral PO_4_ groups, which were also observed in the Bis-2 monomer [37].

The FTIR spectrum of the annealed MCP showed no presence of organic groups, as there were no absorption peaks observed at 1720 cm^−1^, 1638 cm^−1^, and 1454 cm^−1^ (corresponding to the organic monomer Bis-2). Instead, all of the characteristic peaks of apatitic phosphate groups were observed. The FTIR of the MCP showed characteristic absorption bands of HAp, evidenced by the presence of PO_4_^3−^ (υ_1_ 963 cm^−1^, υ_3_ 1020 and 1072 cm^−1^, υ_4_ 603 and 568 cm^−1^), whereas that of annealed MCP showed absorption peaks at 1035 cm^−1^, 956 cm^−1^, 944 cm^−1^, and multiple peaks in the 555–604 cm^−1^ region, indicative of apatite structure. The newly formed peaks at the 940 cm^−1^ region may be attributed to the partial thermal degradation of the apatitic structure to a tricalcium phosphate (TCP) structure. This proves the thermal degradation of both the carbonaceous group and the organic functional group upon annealing at 600 °C for 4 h. The organic, volatile part of the MCP was calculated by weight difference to be 42% of the material. The Ca/P ratio of the heat treated MCP was found to be 1.46, which also indicated a possible decomposition of the MCP to TCP on annealing.

### 3.2. Physical Properties

The translucency parameters (TP) of the experimental composites are presented in Table 2. When measured with a specimen of 2 mm thickness, the 3 wt.% MCP composite presented the highest TP value of 8.6, followed by the composite with 6 wt.% MCP (8.4) and the control specimen without MCP (8.4). The composite with 20 wt.% MCP showed the lowest TP value of 5.4. The high amount of filler in the 20 wt.% MCP composite reduced the TP value. The addition of MCP in the range of 1–6 wt.% did not change the translucency of the composite.

**Figure 2:**
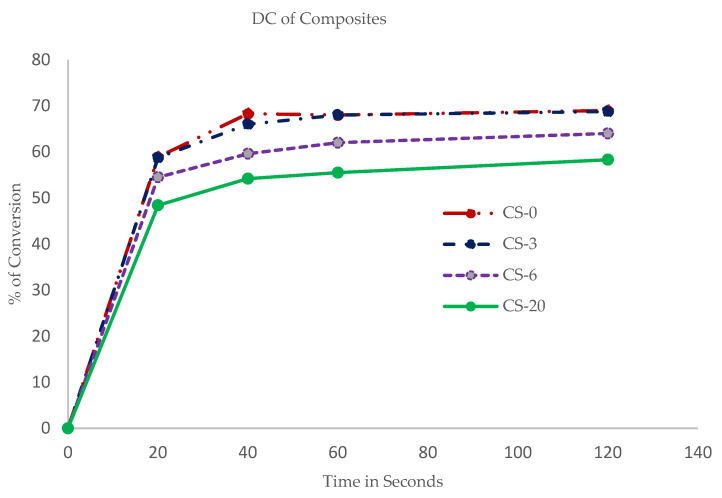
Degree of conversion of composites with respect to time of irradiation shows decreasing conversion rates with increasing percentages of MCP.

The mean degree of conversion (DC), measured at the bottom surface of the specimens as a function of irradiation time, and the amount of the functional MCP filler, is shown in Figure 2. The DC values of the tested specimens (N = 3) were calculated based on the peak area of the C=C group (1638 cm^−1^) of the starting material, represented as 0% of conversion, versus the peak area of the polymerized specimen. The control composite (CS-0) and 3 wt.% MCP composite (CS-3) showed the highest DC values, in the range of 69, and are not statistically different. The DC of the specimens decreased with the amount of added MCP in the specimen. The specimen with 20 wt.% MCP showed the lowest DC at 58%.

The mean ISO depth of cure values are also presented in Table 2. The ISO depth of cure of the composites ranged from 2.48 to 2.66 mm. The CS-3 composite presented a slightly higher depth of cure (2.65 mm) compared to the control composite with no MCP (2.62 mm). The depth of cure for CS-6 was 2.58 mm, and the lowest depth of cure value of 2.48 mm was for the specimen with 20 wt.% MCP.

The mean flexural strength (FS) values of the composites are also presented in Table 2. The specimen with CS-3 presented the highest flexural strength at 106 MPa. The flexural strength values observed for the control specimen and the CS-6 specimen were not significantly different, while the specimen CS-20 had the lowest flexural strength value of 92.6 MPa.

Finally, the deflection at break of the specimens is also presented in Table 2. The mean deflection at break for the specimens containing MCP was higher than those of the control specimen (no MCP). The highest deflection at break, 0.75 mm, was for the specimen containing 6 wt.% MCP (CS-6); however, it was not statistically different from that of the specimens with CS-3 (0.74 mm) and CS-20 (0.72 mm). The deflection at break value for the control composite with no MCP was 0.58 mm.

### 3.3. Calcium and Phosphate Ion Release

**Figure 3:**
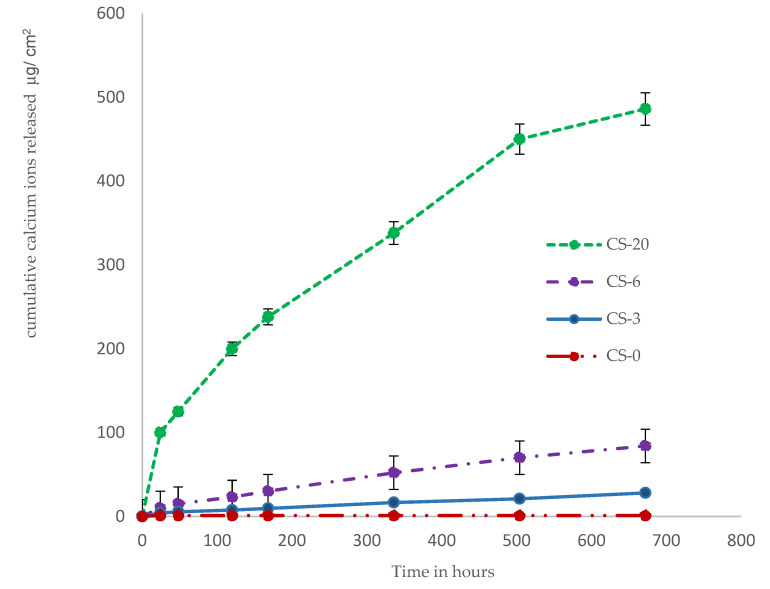
Cumulative release profile data of calcium ions from the composite specimens show escalating release of calcium ions with increasing percentages of MCP.

**Figure 4:**
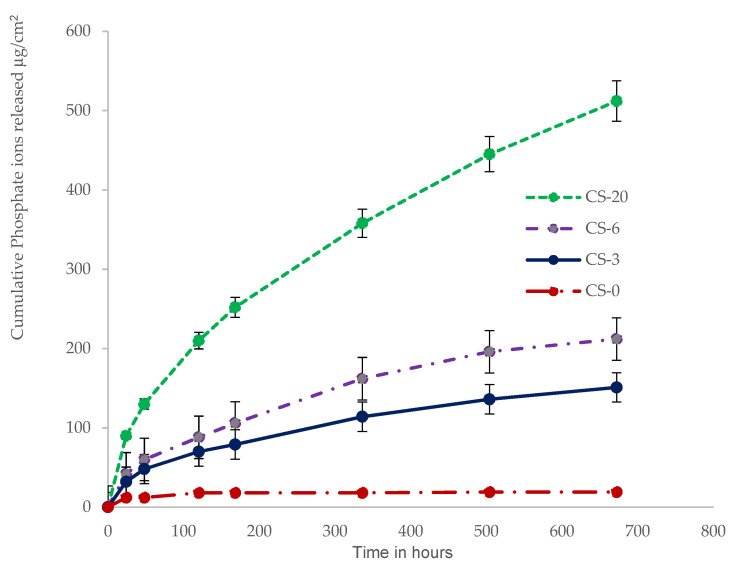
Cumulative release profile data of phosphate ions from the composite specimens show escalating release of phosphate ions with increasing percentages of MCP.

**Figure 5:**
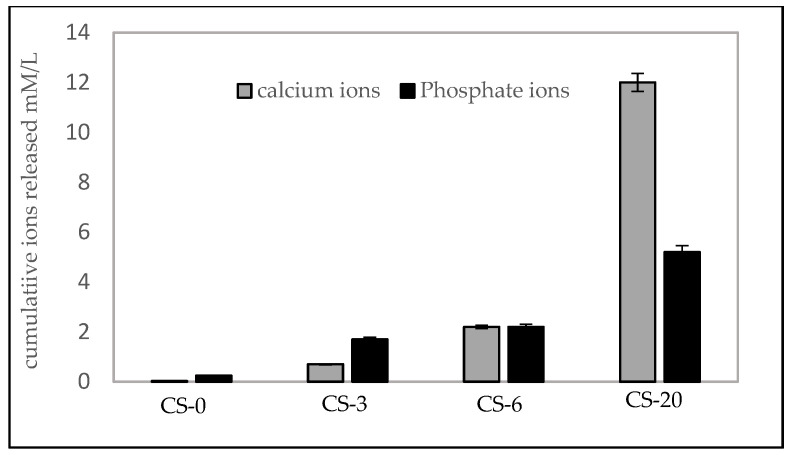
Cumulative ions (mM/L) released from the composite specimens during four week.

Figure 3, Figure 4 and Figure 5 show the cumulative release of calcium ions and phosphate ions from the composite materials as a function of time for the four weeks of the study. Specimens containing MCP showed a continuous release of calcium and phosphate ions over the four-week period. The specimen containing CS-20 showed the highest amount of calcium and phosphate release. The cumulative calcium release from the specimen with 20 wt.% MCP was 12 mM/L or 486 µg/cm^2^, and the cumulative phosphate ion release from that specimen was 5.2 mM/L or 500 µg/cm^2^. The control composite released the lowest amount of calcium and phosphate ions; those primarily originated from the inorganic glass fillers.

The release of calcium and phosphate ions from the various composite materials was directly correlated to the amount of MCP in the material. The relative molar mass release of calcium ions to phosphate ions decreased as the percentage of MCP filler in the composite decreased.

### 3.4. Bioactivity and Apatite Formation

This experiment demonstrated a direct relationship between the hydroxyapatite formation on the surface of the specimens, the percentage of MCP in the specimens, and the number of days of storage in PBS. After 21 days of storage in PBS, all the specimens showed at least partial hydroxyapatite aggregates on this disc surface. As the percentage of MCP increased, there was a dramatic increase in the development of calcium phosphate crystals on the surface of the specimens. The calcium and phosphate contents of the specimens before immersion in PBS were 0.73% and 0.65%, respectively.

**Figure 6:**
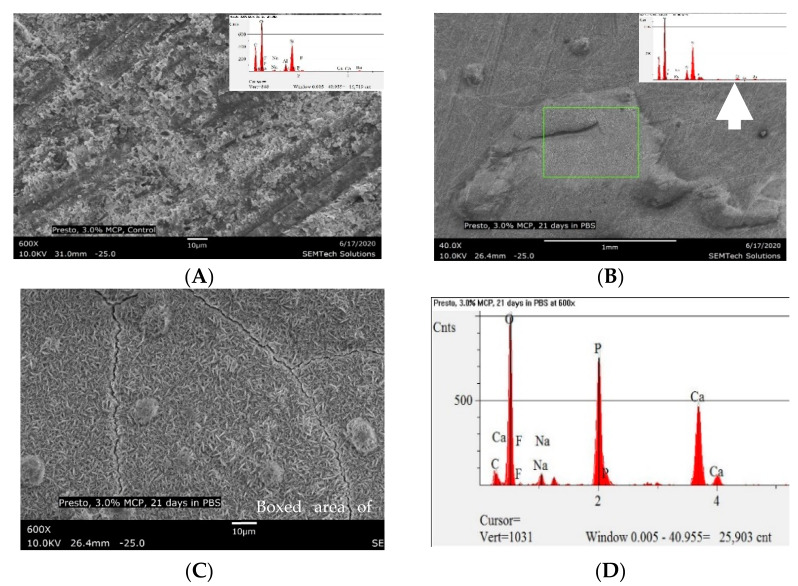
(**A**) SEM micrograph and EDS spectrum of the experimental composite with 3 wt.% MCP (CS-3) before immersion in PBS (600×); and (**B**) SEM micrograph and EDS of CS-3 after 21 days of storage in PBS. 6B shows two areas of calcium phosphate precipitation, a light area (box) and a dark area; (**C**) SEM image of the light boxed area of CS-3; (**D**) EDS of (**C**) indicates well-developed calcium phosphate (CaP) crystals.

The SEM micrographs and EDS of specimens containing 3 wt.% MCP (CS-3), before and after immersion in PBS for 21 days, are presented in Figure 6A–D. These SEM (6B) showed two distinctive and approximately equal areas of crystal growth: (1) light regions covered with aggregates of calcium phosphate and with the potential to serve as nucleation sites for further calcium phosphate deposition; and (2) dark areas where a lower increase in calcium phosphate was observed. The EDS analysis of the light areas showed fully developed calcium phosphate crystals with a high mean Ca:P ratio and no peaks originating from the substrate polymer disk. This indicates extensive growth of apatite in those regions. The EDS analysis of the dark areas showed a five-fold increase in calcium and phosphate ion concentrations compared to the control specimen, much lower than the light areas. The dark areas indicate immature calcium phosphate growth (Table 3).

**Figure 7:**
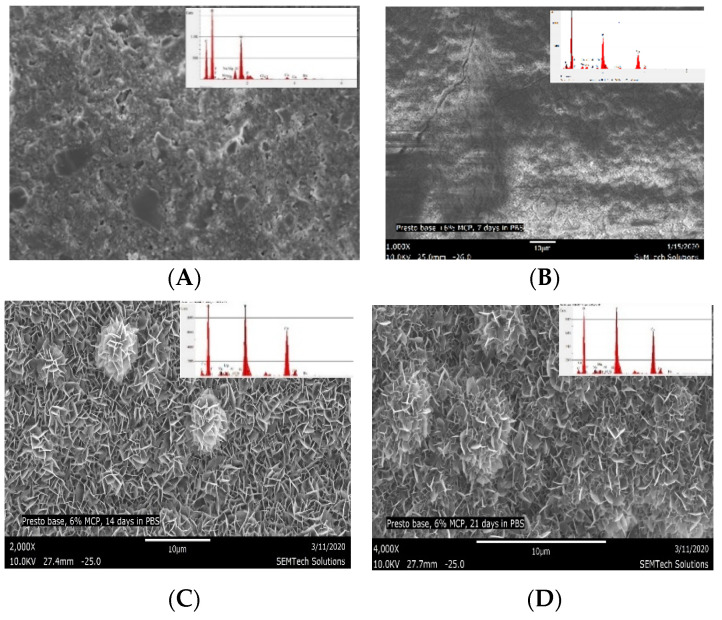
(**A**) SEM micrograph and EDS of the specimen containing 6 wt.% MCP (CS-6) before immersion in PBS; (**B**) after 7 days, (**C**) after 14 days, and (**D**) after 21 days immersion in PBS, showing good surface precipitation of calcium phosphate. The reduced quality of the micrograph of the 7 day specimen may be attributed to the short range order of the amorphous CaP and the accidental inclusion of water (1000×).

**Figure 8:**
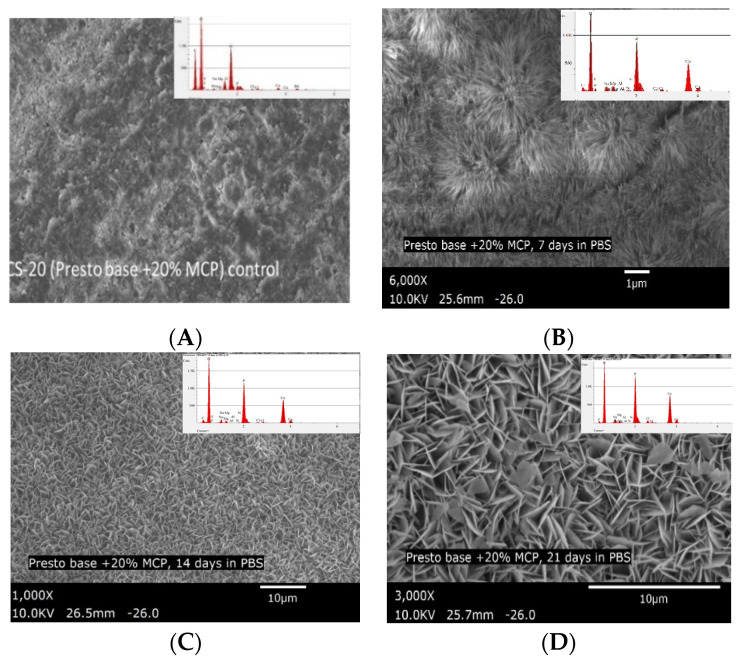
(**A**) SEM micrograph of specimen containing 20 wt.% MCP (CS-20) before immersion in PBS, and SEM micrograph and EDS of specimen after 7 days (**B**); 14 days (**C**); and 21 days (**D**) of immersion in PBS.

In-order to understand the biomineralization potential of MCP as a functional filler, experimental specimens containing even higher percentages of MCP were studied. Specimens with 6 wt.% MCP, after 7 days storage in PBS, showed the entire surface of the specimen disc covered with calcium phosphate nucleation sites (Figure 7A). The EDS after 7 days storage in PBS showed calcium-phosphate ratios ranging from 1.10 to 1.40, suggesting the formation of calcium deficient hydroxyapatite [38]. At 14 days and 21 days of storage in PBS, well-developed hydroxyapatite could be seen throughout the surface of the specimen (The calcium and phosphorous elemental concentrations obtained by EDS are presented in Table 4. SEM and EDS of control specimens and 20 wt.% specimens images are presented in the Figure 8 (see Supplementary file, Figures S3 through S6).

## 4. Discussion

### 4.1. Methacrylated Calcium Phosphate

It is known that the crystallinity and solubility of calcium phosphate can be controlled by adding suitable additives to the reactants [39]. It is also known that the addition of organic or polymeric materials serves to stabilize calcium and phosphate, as these prevent agglomeration and control crystallite growth [22]. The Ca/P compositional ratio of 1.24 suggested that the MCP powder had a compositional formula similar to that of amorphous calcium phosphate [40]. The examination of XRD pattern indicated that the synthesized MCP powder was poorly crystalline and amorphous. The FTIR spectrum of the MCP was very complex due to the presence of multiple P-O absorption bands originating from the Bis-2 monomer. Based on the spectral data and XRD analysis, it can be suggested that the crystal growth of hydroxyapatite (by the reaction of calcium and PO_4_^3−^ ions) was regulated by the bulky organic phosphate group. The linkage of the bulky organic phosphate to the divalent calcium ions occurred through an ionic bonding. In contrast to amorphous calcium phosphate (ACP), MCP exhibited good stability. Compared to crystalline calcium phosphates, amorphous calcium phosphate exhibits greater bioactivity, and has been shown to have better adhesion to periodontal ligament cells [40]. The incorporation of the organic monomer, Bis-2, as a functional reactant to synthesize MCP creates better solubility and stability for the calcium phosphate. MCP has greater solubility compared to the commercial grade HAp or the commercial grade dicalcium phosphate [41]. Furthermore, MCP has moderate solubility in hydrophilic monomers, which enables it to generate a homogeneous phase that can act as a translucent, esthetic, mono-block composite.

### 4.2. Physical Properties

The optical and physical properties of dental composites are highly influenced by the composition of the resin matrix and fillers. Variables include: (i) the resin matrix type and its amount; (ii) the size, shape, and amount of inorganic fillers, (iii) the interaction of the resin matrix and the fillers; and (iv) the amount and type of the photo-initiator system [42,43]. Translucency and opacity have been viewed as vital properties of esthetic dental composite resins. They are the indicators of the quality and quantity of the reflected light, and they have a profound effect on the cure efficiency [44], i.e., the degree of conversion and the depth of cure. It is reasonable to assume that a highly translucent, resin-based composite has a higher degree of conversion, transmits light deeper into the material, and has a greater depth of cure [45] than a less translucent material.

The translucency of composite formulations is highly dependent on the refractive index match of the filler phase and the resin phase [46]. A typical dental composite consists of resin mixtures based on the aromatic monomer 4-bisphenol A-diglycidyl ether dimethacrylate (Bis-GMA), which has a refractive index nD 1.56 and a refractive index of 1.53 when bis-GMA is coupled with an aliphatic diluent or an urethane dimethacrylate monomer system (UDMA nD 1.48). The refractive index of strontium silicate glass is 1.48–1.51, whereas the refractive index of barium glass is 1.51–1.53. Composites appear opaque when there is a large mismatch between the filler and resin refractive indices. Hydroxyapatite (HAp) has a refractive index of 1.63 and the incorporation of HAp fillers reduces the translucency of composite formulations. Therefore, composites containing HAp fillers have drawbacks for forming esthetic dental composites.

The degree of conversion is an important tool to determine the final physical, mechanical, and biological properties of dental composites. The correlation between the degree of conversion and the mechanical performance of photopolymerized dental composites has been well studied [43]. The degree of conversion of a dental composite resin determines the success of direct restorations: (1) greater monomer to polymer conversion corresponds to superior mechanical properties; and (2) greater conversion may result in less uncured, potentially leachable, monomers in the composite [47].

The depth of cure of dental composites is clinically relevant and is defined as “the thickness of a resin that may be converted from a monomer to a polymer, under a specific light curing condition” [48]. Depth of cure serves as a reference for placing the material in increments, without compromising the physical and biological properties of the material. Similar to translucency and degree of conversion, depth of cure is highly influenced by the resin matrix, the photo-initiator system, and the nature of the filler.

In this study, to investigate the effect of the added MCP filler on translucency, degree of conversion, and depth of cure, all composite formulations contained the same resin composition, photo-initiator system, inorganic fillers, and shade. It was found that replacing some of the inorganic filler with an equal amount of MCP improved the translucency parameter, increased the depth of cure, and showed a similar degree of conversion over that of a control composite without MCP. The higher translucency parameter and depth of cure of a composite with 3 wt.% MCP was attributed to the higher interaction and solubility of the MCP filler with the resin phase. The composite with 6 wt.% MCP exhibited a lower mean degree of conversion of 64% and slightly lower depth of cure, but had a similar mean translucency parameter as the control composite with no MCP. On the other hand, composite with 20 wt.% MCP showed the lowest mean value for translucency parameter (5.5), degree of conversion (58%), and a mean depth of cure (2.48 mm). The low depth of cure of CS-20 may be attributed to the higher paste viscosity of the formulation. All tested composites have acceptable depths of cure for clinical application.

The mean flexural strength of the composites with MCP filler was tested by a three-point ISO 4049 method. Composite with 3 wt.% MCP presented the highest flexural strength (106 MPa), which was slightly higher than that of the control composite (98.2 MPa). The composite with 6 wt.% MCP showed slightly higher mean flexural strength compared to the control composite, but the difference was not statistically significant. The composite with 20 wt.% MCP showed the lowest mean flexural strength of 92.6 MPa. The lower values were attributed to the lower degree of conversion due to the high paste viscosity and reduced filler-resin interaction. All composite formulations exhibited good toughness values, which were evidenced by high flexural strength and high deflection at break. The high toughness of the dental composites studied in the present work is attributed to the incorporation of the rubberized urethane in the resin matrix of the composite formulation. Composites with higher toughness and flexural properties enhance the fatigue strength and longevity of dental restorations [26,49]. The high flexural strength and toughness of the 3 wt.% MCP specimens was further confirmed by their low wear and high polishability [41]. The reduction in the physical and mechanical properties of the composites with the incorporation of MCP in the range 6 wt.% or more, partially rejects the null hypothesis.

### 4.3. Ion Realease and Bioactivity of Composites

The ion releasing properties of the experimental composites with MCP were studied in distilled water over a period of four weeks. The ion release data is presented as micrograms of ions released per cm^2^. The release of calcium and phosphate ions is highly influenced by the nature of the resin matrix and the amount of the functional calcium phosphate filler, MCP. The hydrophilic character of the resin matrix plays a significant role in the ion release properties of the experimental composites, and is impacted by the addition of a small amount of phosphate monomer (Bis-2) to the resin matrix. The release of calcium and phosphate ions occurs after the interaction of composite with water or saliva in the oral environment. The release of calcium and phosphate ions from the dental composites was directly correlated to the amount of MCP in all experimental composites. Sustainable releases of calcium and phosphate ions were observed in all experimental composites with MCP fillers over a period of four weeks. Dental composites that release adequate amounts of calcium and phosphate ions have been demonstrated to achieve dentin lesion remineralization [50]. In an earlier study, we reported that a 3 wt.% MCP composite released and recharged fluoride, calcium, and phosphate ions [41]. Composite with 3 wt.% MCP released adequate amounts of calcium and phosphate ions and facilitated hydroxyapatite precipitation on the surface of the specimen when stored in PBS. The present study also showed a direct correlation between the amount of crystalline calcium phosphate precipitated on the surface of the specimen and the length of time the specimen was stored in the PBS.

The biomineralization pathway of MCP containing composites is different from that of bioglass 45S5 or that of calcium-silicate based dental products. In bioglass 45S5 and calcium-silicate based products, such as TheraCal (Bisco, Schaumburg, IL, USA) and MTA (Septodont, Saint-Maur-des-Fossés, France), there is a rapid release of soluble ionic species (Ca^+^ and Si^+^ ions) from the glass; a polycondensation reaction of surface silanols to generate a large number of heterogeneous nucleation sites; and crystallization of biologically reactive hydroxyl carbonate apatites in contact with a phosphate-containing medium (saliva) [51]. In MCP containing composites, the presence of the ionic resins (Bis-2) plays a crucial role in the biomineralization of calcium phosphate and mediates the stabilization of the inorganic calcium phosphate nanocrystals. Based on the observation of higher bioactivity in MCP containing composites, even at a lower weight percentage in the material, one could suggest that the biomineralization pathway of the MCP containing composites proceeds through a matrix–particle-mediated process assisted by the acidic phosphate monomer present in the composite.

The role of the experimental composite’s polyionic resin matrix on the biomineralization process can be further understood by application of various theories proposed by researchers [8,52,53]. Additionally to be considered are: (1) the role of phosphoproteins in the biomimetic templating and stabilization of calcium phosphates [54]; (2) the role of non-collagenous protein (NCP) analogues, such as phosphonic acid and polyacrylic acid, in bio-mineralization [55]; and (3) the role of bio-composites of calcium deficient hydroxyapatite and amino acids [56]. The authors speculate that the ionic phosphate groups in the experimental composite resin matrices highly influenced the growth of calcium phosphate crystals and the bioacivity of the composites. The enhanced bioactivity of the experimental composites is further explained by the addition of seed particles of MCP, which upon reaction with the phosphate resin, form calcium deficient hydroxyapatite nucleation sites for the growth of biological calcium phosphate crystals [57,58].

It was noted that highly crystalline calcium phosphate was formed on the surface of composites containing 6 wt.% MCP after 14 days of immersion in PBS. This is evidenced in the FTIR spectrum by the characteristic absorption peaks of crystalline calcium phosphate at 1032 cm^−1^, 963 cm^−1^, 603 cm^−1^, and 563 cm^−1^ (see supplemental doc). It is also evidenced in the EDS data (Table 4), which indicates the bio-mineralization of CaP is going through an amorphous calcium phase, with a calcium phosphate ratio less than 1.40, on the way to a highly crystalline HAp, with a Ca:P ratio of 1.6, as reported by various researchers.

Calcium phosphate fillers without polymerizable vinyl or methacrylate functional groups lack chemical interaction with the resin phase; their interactions are solely mechanical in nature. The presence of a pendant, polymerizable, methacrylate group in MCP has the advantage of covalently attaching the MCP fillers to polymer resin composites by taking part in free-radical polymerization with vinyl- or methacrylate-functional co-monomers. This chemically modified reinforcement of calcium phosphate creates a more intimate contact with the resin phase, resulting in a dental material that can readily transfer its loads between its resin and filler phases [10,22,23,24]. The superior mechanical properties of the experimental composites with MCP were attributed to the better chemical integration of MCP with the resin phase. Though the in vitro bioactivity studies of MCP-containing esthetic dental composites look very promising, clinical studies are necessary to determine the ability of these composites to prevent secondary caries and microleakage.

## 5. Conclusions

Esthetic dental composites containing varying amounts of methacrylate functional calcium phosphate were investigated for their translucency, degree of conversion, and mechanical properties. The incorporation of MCP as a bioactive filler in esthetic dental composite formulations and its ability to promote precipitation of hydroxyapatite on the surfaces of those composites was evaluated. It was found that there is a direct correlation between the amount of MCP in a composite formulation and the deposition of hydroxyapatite on the material’s surface. The bioactivity of MCP, and its role in an ionic resin to create a nucleation template, was investigated. Methacrylate-functionalized calcium phosphate (MCP) enables the creation of highly mineralized, durable, polymer-based composites with mono-functional, di-functional, or multi-functional monomers.

## 6. Patents

The patent “Stabilized calcium phosphate and methods of forming same” US patent 10,219,986 dated 03-05-2019 resulted from the work reported in this manuscript.

## Figures and Tables

**Table 1:** Composite Formulations.

Sample	TFC%	Resin Matrix %	MCP%	CQ%
Control Comp, 0% MCP (**CS-0**)	69.5	30.0	0.0	0.08
Comp + 3 wt.% MCP (**CS-3**)	69.5	30.0	3.0	0.08
Comp + 6 wt.% MCP (**CS-6**)	70.4	29.0	6.0	0.08
Comp + 20 wt.% MCP	74.4	25.5	20.0	0.08

TFC%: total filler content % includes barium/strontium alumino silicate, silica, and MCP.

**Table 2:** Physical Properties of Composites.

Sample	Degree of Polymerization %	Translucency Parameter (TP)	Depth of Cure (mm)	Flexural Strength (MPa)	Deflection at Break (mm)
CS-0	69 (1.3)	8.4 (0.26)	2.62 (0.01)	98.2 (2.8)	0.58 (0.02)
CS-3	69 (2.2)	8.6 (0.18)	2.65 (0.01)	106 (3.2)	0.74 (0.03)
CS-6	64 (2.8)	8.4 (0.24)	2.58 (0.02)	99.4 (2.9)	0.75 (0.03)
CS-20	58 (3.2)	5.5 (0.38)	2.48 (0.02)	92.6 (3.8)	0.72 (0.02)

**Table 3:** EDS analysis of calcium and phosphorous atom ratios observed on the surface of CS-3, before and after immersion in PBS for 21 days.

Material	Calcium (Ca) %	Phosphorous (P) %	Ca:P Ratio
3 wt.% MCP (CS-3) before immersion in PBS (Figure 6A)	0.73	0.65	1.12
CS-3 after immersion in PBS for 21 days. Light area indicates extensive CaP precipitation (See Figure 6B–D)	31.26	19.42	1.61
CS-3 after immersion in PBS for 21 days. Dark area indicates mild CaP precipitation (See Figure 6B)	2.75	2.22	1.24

**Table 4:** EDS analysis of calcium and phosphorous atom ratios observed on the surface of CS-6 and CS-20, before and after immersion in PBS for 21 days.

Material	Calcium %	Phosphorous %	Ca/P Ratio
Composite with 6% MCP (CS-6) control (not immersed in PBS) (6000×)	2.60	2.50	1.03
CS-6 after 7 days immersion in PBS (6000×)	29.81	21.79	1.37
CS-6 after 14 days immersion in PBS (6000×)	31.64	20.39	1.55
CS-6 after 21 days immersion in PBS (6000×)	30.52	19.63	1.56
Composite with 20% MCP (CS-20) control (not immersed in PBS) (6000×)	8.40	6.90	1.22
CS-20 after 7 days immersion in PBS (6000×)	27.07	20.25	1.34
CS-20 after 14 days immersion in PBS (6000×)	30.12	19.62	1.54
CS-20 after 21 days immersion in PBS (6000×)	32.49	20.31	1.60

## Data Availability

The data can be found in website www.pulpdent.com (accessed on 20 February 2020) (Activa Presto) and all SEM and EDS images are available as jpeg, on request.

## Supplementary Materials

The following are available online at https://www.mdpi.com/article/10.3390/polym13132095/s1, Figure S1: XRD of MCP powder. Figure S2: FTIR spectrum of precipitated calcium phosphate. Figure S3: SEM and EDS of CS-0 after immersion in PBS. Figure S4: SEM and EDS of CS-3. Figure S5: SEM and EDS of CS-6 Figure S6: SEM ad EDS of CS-20, after immersion in PBS.
