# Supplementary material for: Experimental Dental Composites Containing a Novel Methacrylate-Functionalized Calcium Phosphate Component: Evaluation of Bioactivity and Physical Properties
Source: Polymers (Basel). 2021 Jun 25;13(13):2095. doi: 10.3390/polym13132095 (PMC8271644; doi:10.3390/polym13132095)
Supplement: Supplementary file 1 [file polymers-13-02095-s001.zip › polymers-1238878-supplementary.pdf]

SUPPLEMENTAL DOC (POLYMERS 2021)

1. **Synthesis of Methacrylated calcium Phosphate (MCP) (A typical procedure)**

The preparation of stabilized calcium phosphate was done using calcium chloride, calcium hydroxide, Bis 2 and phosphoric acid (made with 70:30 calcium chloride and calcium hydroxide). In a 1Gal reactor equipped with thermometer, mechanical stirrer and pH meter, 100 g of calcium chloride dihydrate (0.68M) and 21.9 g calcium hydroxide (0.29M) were dissolved in 180 mL distilled water and placed in a water bath at a temperature of about 45° C. The pH of the solution was about 5. To this solution, 33.8 g H_3P0_4 (80%, 0.28M) dissolved in 60 mL distilled water was added gradually under constant mechanical stirring. After the addition, the mixture was stirred f for another 30 min. To this mixture, 89.6.g Bis -2 (0.28M) dissolved in 100 mL distilled water was added gradually. The reaction was stirred for 1h at 40-45°C. To this ammonia solution was added until calcium phosphate was precipitated and the pH of the solution was maintained at pH 10. The suspension was stirred for an additional 6 h at 45°C. The resulting product was filtered on Whatman filter paper, washed with excess water until the product was free of ammonia and dried at about 45°C for 4 days. The white powder was obtained in good yield. 148 g (92% of theoretical yield). The FTIR spectrum of the material shows peaks corresponding to an ester (C=O—) peak at 1730 cm^{-1} and corresponding to a vinyl (C=C) peak at 1638 cm^{-1} in addition to peaks of calcium phosphate (—CaPO), alkyl (C—H) and alkyloxy (C—O) groups. This indicates that the calcium phosphate is stabilized by Bis 2. XRD pattern of the material shows a pattern corresponding to high lattice disorder. The material also exhibits much higher solubility than the control, the SEM shows calcium phosphate particles of irregular shapes and low particle sizes (mostly less than 10 nm).

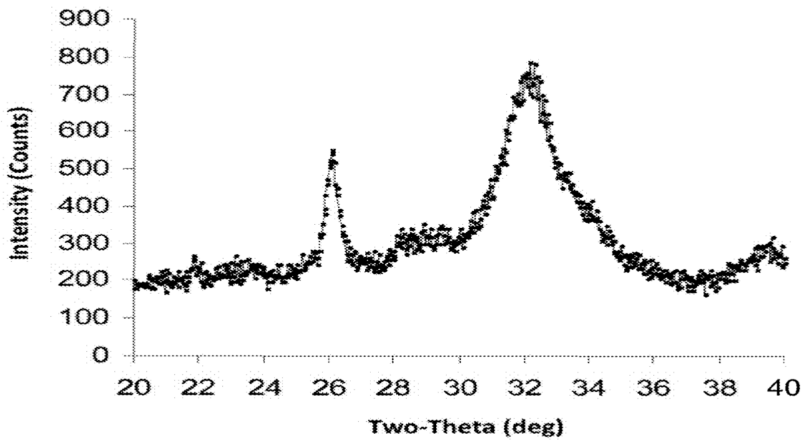

Fig. S1. XRD of methacrylated calcium phosphate (MCP) powder, showing amorphous characteristic of MCP.

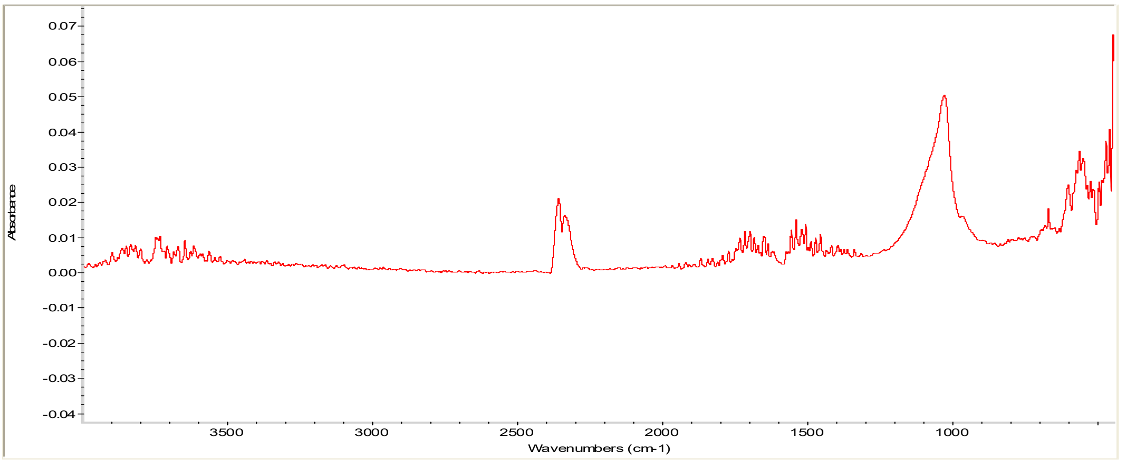

Fig. S2. FTIR spectrum of precipitated calcium phosphate from the surface of Presto composite after 21 of storage in PBS peaks 963 cm^{-1}, 1034 $cm^{-1,}$ which are characteristic of hydroxyapatite peaks.

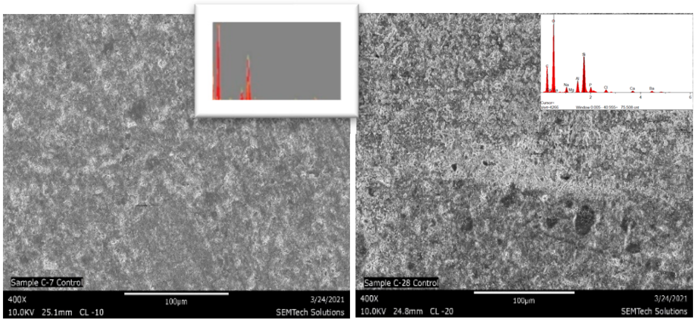

Fig. S3. Composite with no MCP after immersion in PBS for 7 days and 28 days respectively, showing no formation of calcium phosphate precipitation.

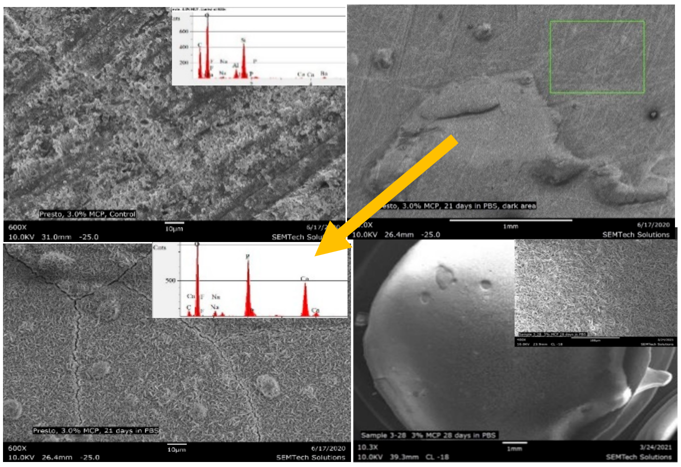

Fig. S4. SEM and EDS of composite specimens with 3 wt.% MCP control specimen (no Immersion PBS) and specimen after 21 days and 28 days of storage in PBS. Specimen with 21 days storage two domain of calcium phosphate precipitation, a thick layer of heavy precipitation and layer of mild precipitation. Specimen showed complete surface precipitation of calcium phosphate on 28 days of storage in PBS.

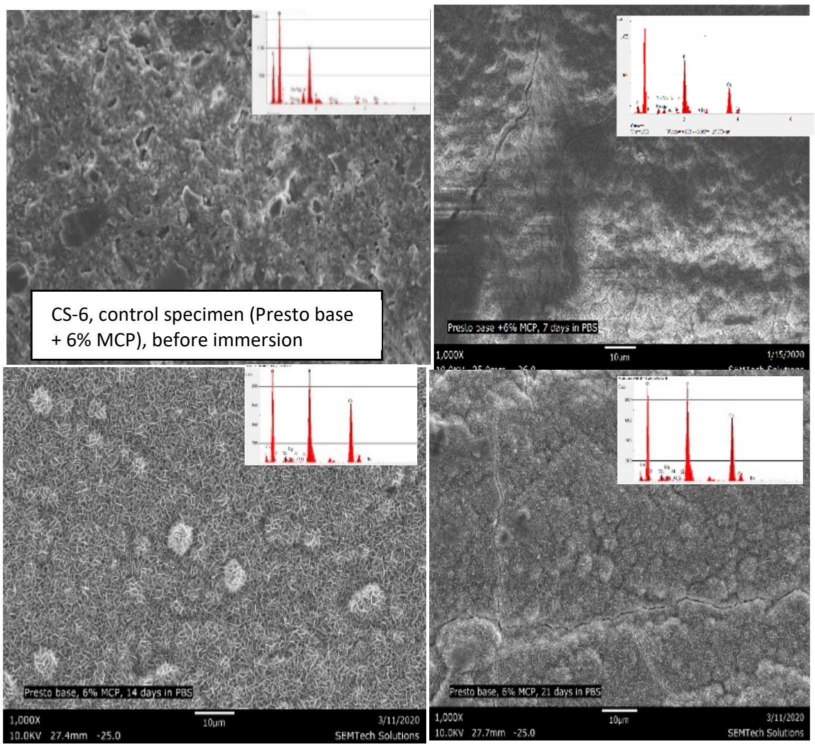

Fig. S5. SEM and EDS of composite specimens with 6 wt.% MCP control specimen (no Immersion PBS) and specimen after 7 days, 14 days and 21 days of immersion in PBS.

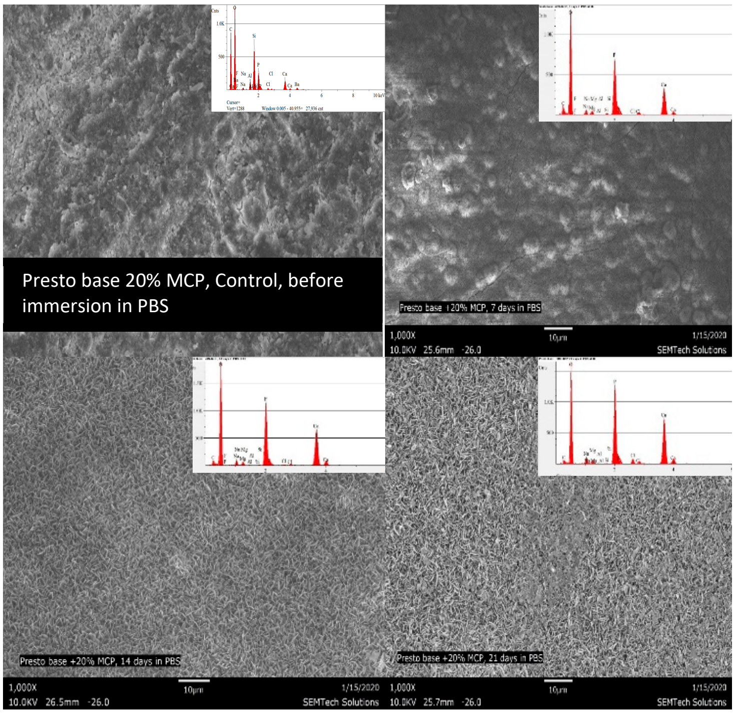

Fig. S6. SEM and EDS of composite specimens with 20 wt. % MCP control specimen (no Immersion PBS) and specimen after 7days, 14 days and 21 days of immersion in PBS.
